# Somatostatin Receptor 2: A Potential Predictive Biomarker for Immune Checkpoint Inhibitor Treatment

**DOI:** 10.3389/pore.2022.1610196

**Published:** 2022-02-21

**Authors:** Aoyun Wang, Yixiao Yuan, Han Chu, Yixing Gao, Zheng Jin, Qingzhu Jia, Bo Zhu

**Affiliations:** ^1^ Institute of Cancer, Xinqiao Hospital, Third Military Medical University, Chongqing, China; ^2^ Chongqing Key Laboratory of Tumor Immunotherapy, Chongqing, China; ^3^ Department of Thoracic Surgery, The Third Affiliated Hospital of Kunming Medical University, Kunming, China; ^4^ Center of Growth, Metabolism and Aging, Key Laboratory of Bio-Resources and Eco-Environment, College of Life Sciences, Sichuan University, Chengdu, China; ^5^ Research Institute, GloriousMed Clinical Laboratory (Shanghai) Co., Ltd., Shanghai, China

**Keywords:** bioinformatics, tumor microenvironment, immune checkpoint inhibitors, somatostatin receptor 2, predictive biomarker

## Abstract

Somatostatin receptor 2 (*SSTR2*), the most abundant receptor of somatostatin (*SST*), possesses immunoreactivity and is altered in many cancers. However, the association between *SSTR2* and efficacy of immune checkpoint inhibitors (ICIs) has not yet been reported. Immunohistochemistry (IHC) information across 20 cancers was collected from the Human Protein Atlas (HPA) and used to analyze the expression of *SSTR2*. Immune signatures collected from public databases, such as BioCarta or Reactome, were used to investigate the association between *SSTR2* and the tumor microenviroment in the Cancer Genome Atlas (TCGA). Data from cohorts treated with ICIs were collected to assess whether *SSTR2* is associated with benefits from ICIs treatment. In the HPA, we found the *SSTR2* IHC-positive rate of 13 cancers to be above 50%. Five types of cancer express *SSTR2* mildly (positive rate: 25%–50%), while the remaining two types of cancer barely stained *SSTR2*-positive (positive rate: 0%–24%). In TCGA analysis, immune cell signatures and immune function pathways were enriched in high *SSTR2* expression groups in most cancers. In each ICIs treated cohort, patients with high *SSTR2* expression experienced numerically superior objective response rate (Braun: 14.8% vs 13.4%, *p* = 0.85; Gide: 69.4% vs 40.5%, *p* = 0.025; Mariathasan: 22.4% vs 16.7%, *p* = 0.233; Miao: 37.5% vs 11.8%; Riaz: 32.0% vs 7.7%, *p* = 0.067) and overall survival (Braun: HR (95%CI): 0.80 [0.62–1.04], *p* = 0.80; Gide: HR (95%CI): 0.61 [0.29–1.30], *p* = 0.20; Mariathasan: HR (95%CI): 0.83 [0.64–1.08], *p* = 0.16; Miao: HR (95%CI): 0.24 [0.086–0.65], *p* = 0.0028; Nathanson cohort: HR (95%CI): 0 [0-inf], *p* = 0.18; Riaz: HR (95%CI): 0.24 [0.086–0.65], *p* = 0.028) than patients with low *SSTR2* expression. In pooled cohort, we found these differences were significant (Pool: 24.6% vs 16.7%, *p* = 0.0077; HR (95% CI): 0.77 [0.65–0.91], *p* = 0.0018). Our results suggest that *SSTR2* is a potential predictive biomarker for response to ICIs.

## Introduction

Immune checkpoint inhibitors (ICIs), which are mainly comprised of anti-programmed cell death (ligand)-1 (*PD-1/PD-L1*) and anti-cytotoxic T lymphocyte-associated antigen 4 (*CTLA-4*) drugs, have revolutionized the therapeutic landscape for many advanced cancers (1–4). However, limited response rate and occasional adverse reactions make it difficult to implement ICIs in clinical practice (5, 6). Biomarkers are helpful in identifying ICIs-sensitive patients, protecting them from unnecessary adverse reactions and reducing financial burden. Thus, further research regarding predictive biomarkers for ICIs is urgently needed (7, 8).

Recent studies have discovered various predictive biomarkers for ICIs, including *PD-L1* immunohistochemistry (IHC), microsatellite instability (MSI), tumor mutation burden (TMB) and multiple gene signatures (9–14). However, there are limitations to their use in clinical practice. For example, some *PD-L1* positive patients were not responsive to ICIs, while those that did not express *PD-L1* were responsive to ICIs (15, 16). Additionally, TMB calculation lacked a standardized formula and unified cut-off value (17). Furthermore, only *PD-L1* and MSI have been clinically validated (18). Thus, finding novel predictive biomarkers is beneficial for the clinical practice of ICIs.

Somatostatin receptor 2 (*SSTR2*), the most abundant somatostatin (*SST*) receptor, is a member of the G protein-coupled receptor family (19). In previous studies, *SSTR2* was proven to be overexpressed in neuroendocrine neoplasms (20, 21). Recent studies show that *SSTR2* is significantly methylated in colorectal cancer (22). *SSTR2* was also proven to be associated with tumorigenesis in gastric cancer and breast cancer (23, 24). Additionally, researchers found that binding of *SST* and *SSTR2* could inhibit immune cells cytokine release and have an effect on the tumor microenvironment (TME) (25, 26). However, the relationship between *SSTR2* and TME and the association between *SSTR2* and prognosis of ICIs have not yet been reported.

In this study, we investigate the expression of *SSTR2* across multiple types of cancer by collecting *SSTR2* IHC data from the Human Protein Atlas (HPA). RNA-seq information from the Cancer Genome Atlas (TCGA) database and immune signatures were used to analyze the underlying mechanism of the effect of *SSTR2* on TME. Then, we collected the mutation and survival information of TCGA patients to investigate the association between *SSTR2* alteration and conventional treatment prognosis. By using collected RNA-seq data and clinical information of patients treated with ICIs, we further investigated the association between *SSTR2* and the efficacy of ICIs treatment.

## Methods

### Public Data Collection

The SSTR2 IHC results were obtained from the HPA (https://www.proteinatlas.org/). The antibody used in IHC was HPA007264, and the further information of antibody was provided in https://www.proteinatlas.org/ENSG00000180616-SSTR2/antibody. The “high,” “medium”, “low”, and “not detected” stain levels were defined by the HPA. Patients RNA-seq data across 33 cancers from the TCGA database were used to explore the underlying mechanism of the effect of *SSTR2* on the immune microenvironment. Mutation information from TCGA was used to investigate the effect of *SSTR2* alteration on prognosis. Because of the possible effects of a physiological barrier on immune cell infiltration LGG, GBM, TGCT, THYM, and UVM were excluded from our study. DLBC and LAML were excluded because they were non-solid cancers.

The clinical and RNA-seq data of one bladder cancer cohort (Mariathasan cohort: *n* = 348), three melanoma cohorts (Gide cohort: *n* = 73; Nathanson cohort: *n* = 9; Riaz cohort: *n* = 51), and two renal cell carcinoma cohorts (Braun cohort: *n* = 311; Miao cohort: *n* = 33) were collected and consolidated to investigate the possible effects of *SSTR2* on ICIs treatment prognosis (27–32). All patients were treated with anti-*PD-1/PD-L1*, anti-*CTLA4*, or a combination of anti-*PD-1/PD-L1* and anti-*CTLA4* drugs. The ICIs treatment efficacy was defined by using Response Evaluation Criteria in Solid Tumors, version 1.1. When patients achieved complete response or partial response, they were considered objective response to ICIs. When patients achieved objective response, or were evaluated keeping in stable disease for longer than 6 months, they were noted responders of ICIs treatment. All patients were divided into an *SSTR2*-high group or *SSTR2*-low group based on the median *SSTR2* expression.

### Gene Set Enrichment Analysis

The immune cell (T cell, central memory CD8 T cell, activated CD8 T cell, effector memory CD8 T cell, type 1 T helper cell, central memory CD4 T cell, activated CD4 T cell, and effector memory CD4 T cell) signatures were collected from public studies (33, 34). The immune functional pathway signatures (interferon alpha/beta signature, T helper pathway, interleukin 15 signature, inflame pathway, interleukin 2 signature, T cytotoxic signature, and T cell receptor activation (TCRA) pathway) were extracted from the BioCarta or Reactome databases. Hallmark gene signatures (hallmark interferon-γ signature and hallmark inflammatory response signature) were collected from the gene set enrichment analysis (GSEA) hallmark gene set (https://www.gsea-msigdb.org/gsea/index.jsp). The GSEA method was described in published research (35). The Pearson correlation test was used to analyze the correlation between *SSTR2* expression and immune signature scores. All signature scores were calculated through the use of a single-sample (ss) GSEA method in R package GSVA (https://www.bioconductor.org/packages/release/bioc/html/GSVA.html).

### Statistical Analysis

The log-rank test and Kaplan–Meier KM method were used to compare overall survival (OS) between the *SSTR2*-high group and *SSTR2*-low group in cohorts treated with ICIs. Univariate Cox analysis was used to define high *SSTR2* expression as protective (0 < HR < 1) or as a risk factor (HR > 1) for prognosis of ICIs treatment. Chi-Squared Test was used to compare objective response rate and responders percentage between *SSTR2*-high expression group and *SSTR2*-low expression group in ICIs treated cohorts with sufficiently high case numbers (case number >40). The results were considered significant when *p* value <0.05. All statistical analyses were performed using R version 4.0.0.

## Results

### Expression of *SSTR2* Across Multiple Cancers

**FIGURE 1:**
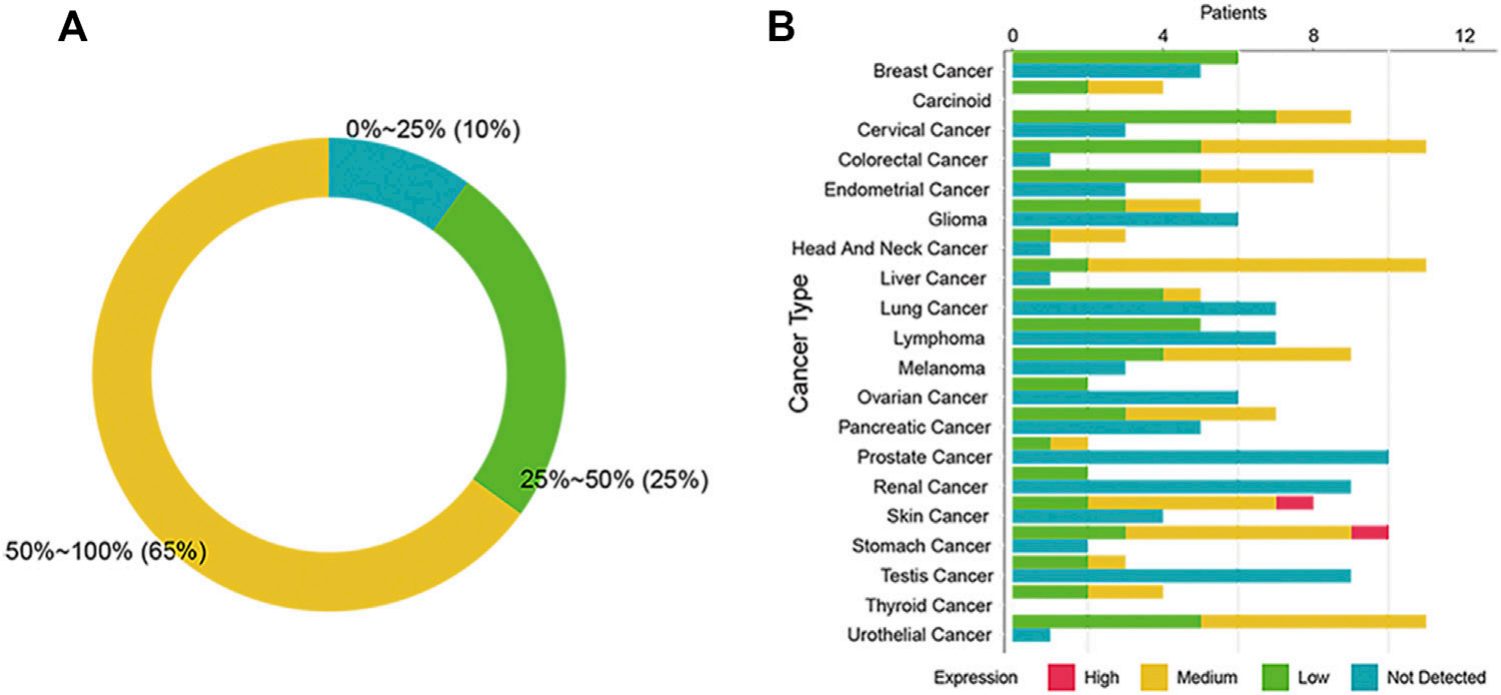
Expression of SSTR2 across 20 cancers in the human protein atlas. **(A)** The summary graph of the SSTR2 immunohistochemistry (IHC) positive rate in the human protein atlas. **(B)** The SSTR2 IHC results of 20 cancers.

To investigate the expression of *SSTR2* in different cancers, we consolidated the IHC data from the HPA. We found the *SSTR2* IHC-positive rate of 13 cancers to be above 50% (Figure 1A). Five cancers express *SSTR2* mildly (positive rate: 25%–50%) and two cancers barely expressed *SSTR2* (positive rate: 0%–24%, Figure 1A). We found that patients with carcinoid and thyroid cancer have the highest positive rate of *SSTR2* IHC among 20 types of cancer (100%, Figure 1B). The cancers with the next highest *SSTR2* IHC-positive rates included colorectal cancer (91.7%), liver cancer (91.7%) and urothelial cancer (91.7%, Figure 1B). Additionally, one stomach cancer patient and one skin cancer patient showed a high staining of *SSTR2* (Figure 1B). The lowest positive *SSTR2* IHC rate was found in prostate cancer (16.7%, Figure 1B). Only prostate cancer and renal cancer *SSTR2* IHC rates were below 25% (Figure 1B). Our findings suggested that *SSTR2* expression varies and is widely distributed across multiple cancer types.

### *SSTR2* is Associated With an Activated Immune Microenvironment

**FIGURE 2:**
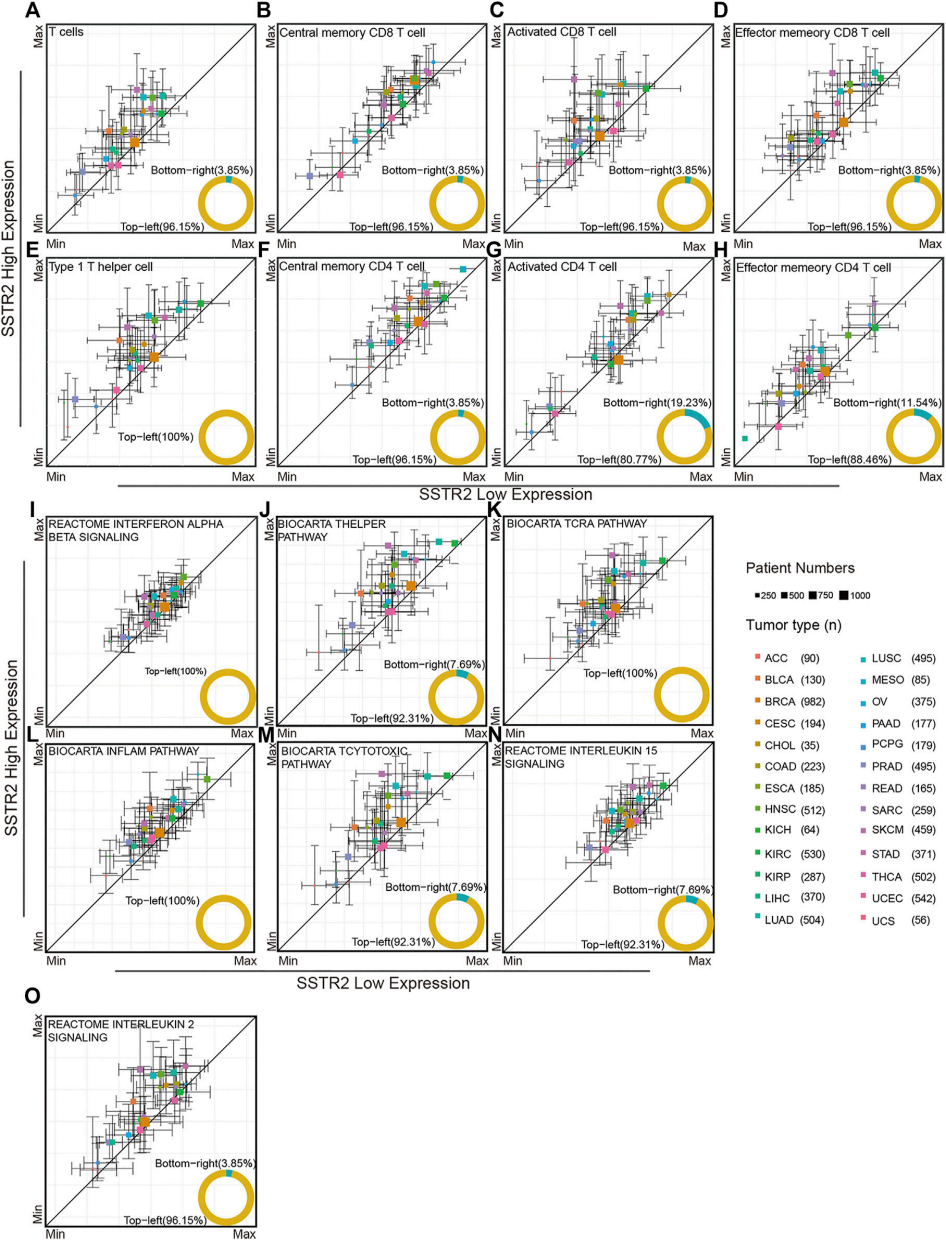
Immune signatures compared between the SSTR2-high group and SSTR2-low group. **(A–H)** Immune cell signatures in SSTR2-high and SSTR2-low groups. **(I–O)** Immune functional pathway signatures in SSTR2-high and SSTR2-low groups. The square point Y-axis positions were the scores median of SSTR2-high group, X-axis positions were the scores median of SSTR2-low group. Bars show 25%–75% scores range of different groups in each cancer.

We investigated the association of *SSTR2* expression and immune microenvironment through TCGA database. By using ssGSEA analysis, we found that T cell signature tends to enrich the tumor microenvironment in *SSTR2*-high groups in most cancers (96.15%, Figure 2A). Then, we analyzed the subpopulation of T cells. The *SSTR2*-high groups have a higher median of central memory CD8 T cell scores, activated CD8 T cell scores, and effector memory CD8 T cell scores in most cancers (central memory CD8 T cell scores: 96.5%; activated CD8 T cell scores: 96.15%; effector memory CD8 T cell scores: 96.5%; Figures 2B–D), suggesting better T cell infiltration may be possible in *SSTR2*-high groups. Type 1 helper cells and CD4 T cells exert a crucial effect on the anti-tumor environment. We also calculated the type 1 helper cell scores and CD4 T cell subpopulation scores of TCGA patients. Median type 1 helper cell scores in *SSTR2*-high groups were higher than those of *SSTR2*-low groups in all 26 cancers (Figure 2E). Additionally, *SSTR2*-high groups of most cancers had higher median central memory CD4 T cell scores, activated CD4 T cell scores, and effector memory CD4 T cell scores (96.15%, 80.77%, and 88.46%, respectively; Figures 2F–H). Our findings suggest that *SSTR2*-high groups may have better immune cell infiltration than *SSTR2*-low groups in various cancers.

We further investigated the immune functional pathway signature distribution across multiple cancers. The interferon alpha beta signature, T helper pathway, and TCRA pathway of *SSTR2*-high groups were higher than those of *SSTR2*-low groups in most cancers (interferon alpha beta signature: 100%; T helper signature: 92.31%; and TCRA pathway: 100%, Figures 2I–K), suggesting that the *SSTR2*-high group has better T cell activation than the *SSTR2*-low group in most cancers. The same results were observed in the inflammatory pathway and T cytotoxic pathway, suggesting that *SSTR2*-high groups showed a stronger inflammatory and cytotoxic immune environment than *SSTR2*-low groups in most cancers (inflammatory pathway: 100%; T cytotoxic pathway: 92.31%, Figures 2L,M). T cells survival in tumor tissue is vital to sustain an anti-tumor response, which relies on the interleukin 15 pathway and interleukin 2 pathway. Our results show that the interleukin 15 pathway appeared to increase in activity in *SSTR2*-high groups in 92.31% of cancers, and the interleukin 2 pathway appeared to increase in activity in *SSTR2*-high groups in 96.15% of cancers (Figures 2N,O). Our findings suggest that better activation and maintenance of cell-mediated immunity may exist in patients with a high expression of *SSTR2*.

**FIGURE 3:**
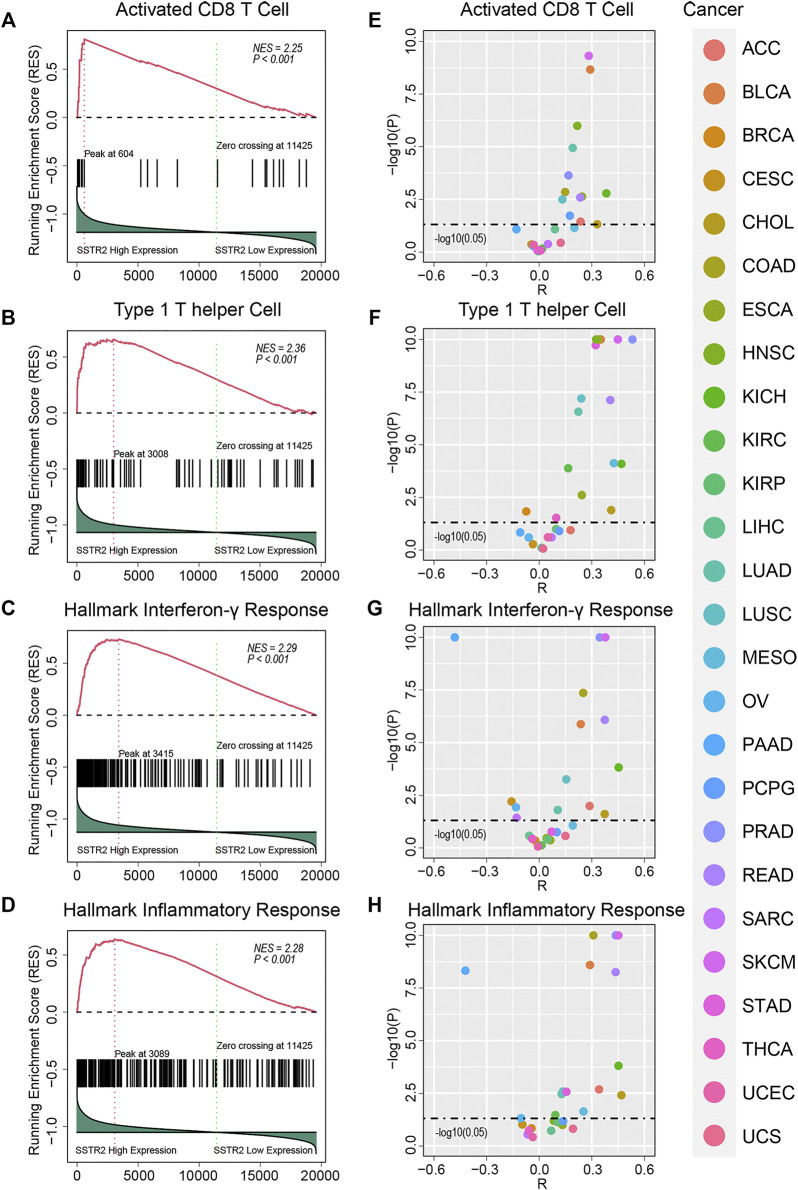
Immune signature enrichment results and correlation between SSTR2 expression and immune signatures scores. **(A–D)** immune signatures enrichment plots in TCGA pooled Q18 cohort. **(E–H)** Correlation summary plots of SSTR2 expression and immune signature scores.

We then performed GSEA analysis to compare the immune microenvironment between *SSTR2*-high patients and *SSTR2*-low patients. We found that immune cell signatures were higher in *SSTR2*-high patients in TCGA pooled cohort (activated CD8 T cell signature: normalized enrichment score (NES) = 2.25, *p* < 0.001; type 1 helper cell signature: NES = 2.36, *p* < 0.001, Figures 3A,B). Immune functional signature results were consistent with those of the immune cell signatures (hallmark interferon-γ signature: NES = 2.29, *p* < 0.001; hallmark inflammatory response signature: NES = 2.28, *p* < 0.001, Figures 3C,D). We then analyzed the correlation between *SSTR2* expression and immune signature ssGSEA scores. We found that *SSTR2* tends to be positively correlated with immune signature scores in most cancers. In BLCA, KICH, LUSC, PRAD and SKCM, *SSTR2* expression is positively correlated with four signatures (Figures 3E–H). Our findings suggest that high *SSTR2* expression is accompanied by an actived immune microenvironment in various cancers.

### *SSTR2* is Not a Prognostic Factor in TCGA Pooled Cohort

We then investigated the effects of *SSTR2* expression and alteration on prognosis in TCGA. We found the average alteration rate of *SSTR2* in TCGA was 1.18% (Supplementary Figure S1). The top five cancer with highest alteration rate were UCEC, COAD, SKCM, READ, STAD (UCEC: 5.85%; COAD: 3.51%; SKCM: 3.43%; READ: 2.19%; STAD: 1.37%; Supplementary Figure S1). Additionally, there was no significant difference between OS in *SSTR2*-high patients and *SSTR2*-low patients (HR (95% CI): 0.94 [0.86–1.03], *p* = 0.20, Supplementary Figure S2). *SSTR2* mutation likely did not affect the prognosis in TCGA pooled cohort (HR (95% CI): 0.77 [0.49–1.19], *p* = 0.22, Supplementary Figure S2). This finding suggests that *SSTR2* is not a prognostic factor in TCGA pan-cancers cohort.

### *SSTR2* is Associated With Prognosis of ICIs Treatment

**FIGURE 4:**
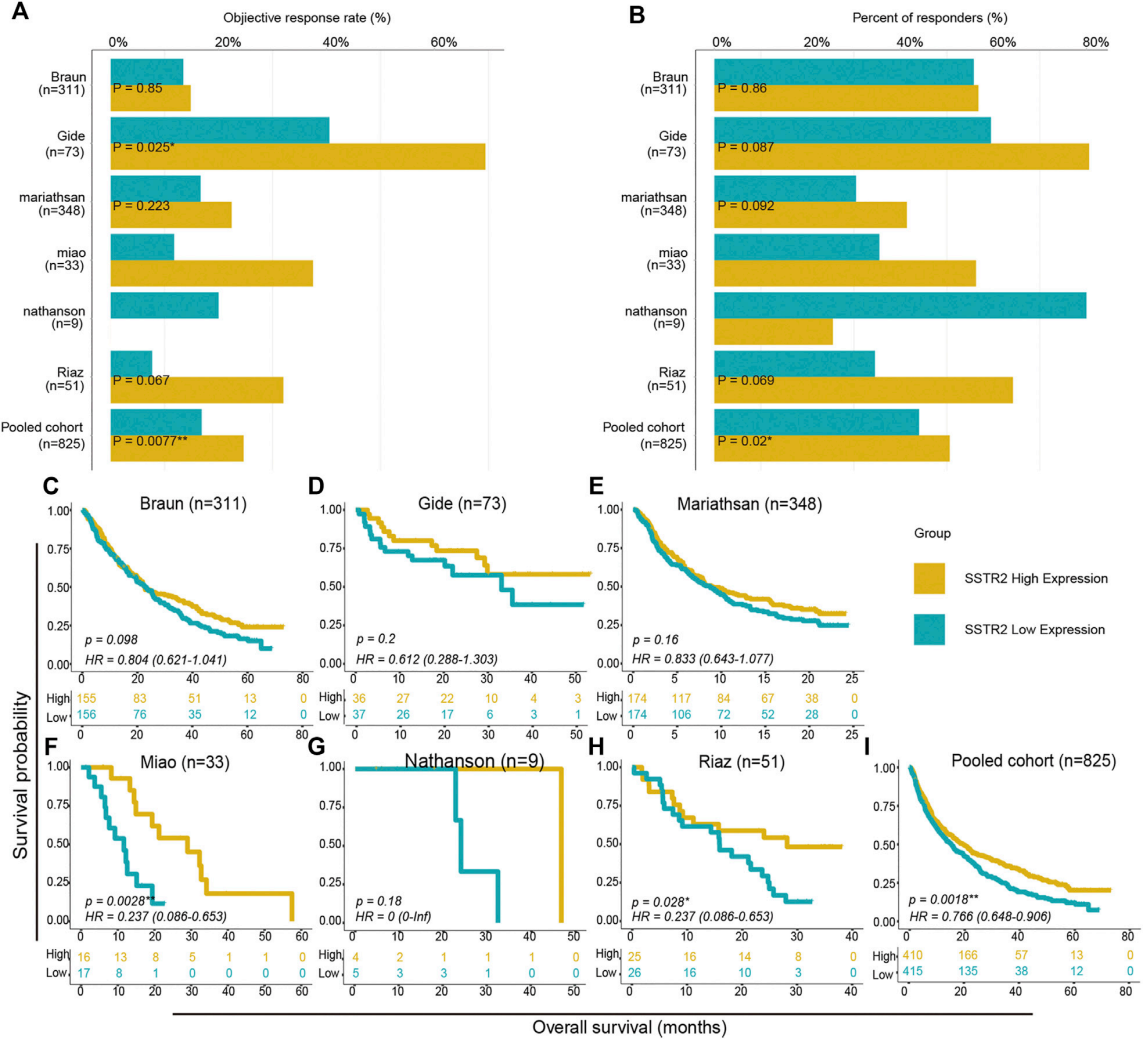
Survival analysis between SSTR2-high groups and SSTR2-low groups in ICIs treated cohorts. **(A,B)** Histogram describing objective response rate and responder percentage. **(C–I)** Kaplan-Meier curves of overall survival (OS) in ICIs treated cohorts comparing patients with high and low SSTR2 expression.

We then aimed to investigate the effect of *SSTR2* expression on the prognosis of ICIs treatment. With the exception of the Nathanson cohort, the objective response rates of *SSTR2*-high groups were numerically higher than those of *SSTR2*-low groups (Braun: 14.8% vs 13.4%; Gide: 69.4% vs 40.5%; Mariathasan: 22.4% vs 16.7%; Miao: 37.5% vs 11.8%; Riaz: 32.0% vs 7.7%; Pooled: 24.6% vs 16.7%; Figure 4A). As Chi-Squared Test showed, in Gide cohort and Pooled cohort, the differences were significant (Gide: *p* = 0.025; Pooled: *p* = 0.0077; Figure 4A). Consistently, the *SSTR2*-high groups in the Braun, Gide, Mariathasan, Miao and pooled cohorts, had numerically higher response rate than the *SSTR2*-low groups (Braun: 54.8% vs 53.2%; Gide: 80.6% vs 59.5%; Mariathasan: 39.7% vs 30.5%; Miao: 56.3% vs 35.3%; Pooled: 51.0% vs 42.7%; Figure 4B), but only in pooled cohort, the result was significant (*p* = 0.02; Figure 4B). These results might suggest that patients with high *SSTR2* expression are more likely to respond to ICIs than patients with low *SSTR2* expression. Thus, We compared the patients OS between *SSTR2* high expression group and *SSTR2* low expression group. We found that patients with high *SSTR2* expression experienced significantly longer OS than patients with low *SSTR2* expression in the Miao, Riaz, and pooled cohorts (Miao: HR (95% CI): 0.24 [0.086–0.65], *p* = 0.0028, Riaz: HR (95% CI): 0.24 [0.086–0.65], *p* = 0.028, Pool: HR (95% CI): 0.77 [0.65–0.91], *p* = 0.0018, Figures 4F,H,I). The same results were observed in the Braun, Gide, Mariathasan, and Nathanson cohorts; however, these results were not found to be significant (Braun: HR (95% CI): 0.80 [0.62–1.04], *p* = 0.80; Gide: HR (95% CI): 0.61 [0.29–1.30], *p* = 0.200; Mariathasan: HR (95% CI): 0.83 [0.64–1.08], *p* = 0.16; Nathanson cohort: HR (95% CI): 0 [0-inf], *p* = 0.18, Figures 4C–E,G). Our findings suggest that patients with high *SSTR2* expression might obtain more benefits from ICIs, such as a higher response rate and longer OS, than patients with low *SSTR2* expression.

## Discussion

The clinical use of ICIs has been proven to result in a better prognosis than conventional treatments in multiple types of cancer. However, the majority of patients do not respond to ICIs. Thus, biomarkers for predicting patients who can benefit from ICIs need to be investigated. In this study, we found that high expression of *SSTR2* is associated with an activated immune microenvironment across multiple cancers. We then analyzed whether *SSTR2* expression was associated with the efficacy of ICIs across multiple types of cancer. Our results suggest that *SSTR2* expression varies among the types of cancer and patients with high *SSTR2* expression could obtain longer OS after ICIs treatment. The *SSTR2*-high groups were also found to have a numerically higher objective response rate and more patients responded to ICIs treatment than in the *SSTR2*-low groups.

*SST*, which is mainly produced by the nervous system and peripheral digestive system, is a strong inhibitory peptide of secretory response of target cells, including inhibition of release of growth hormone, gastro-intestinal hormones and pancreatic enzymes (36). The function of *SST* has been profoundly investigated in previous studies using its receptors (*SSTR1-SSTR5*) as mediators (25). Among all *SST* receptors, *SSTR2* is the most abundant (19). Additionally, *SSTR2* is expressed in human pancreatic tissue, but could be loss in pancreatic cancers and derived cell lines(37–39). Previous studies have demonstrated that the combination of *SST* and *SSTR2* could inhibit cytokine release from immune cells (26). In colorectal cancer, *SSTR2* was shown to be significantly methylated, which results in *SSTR2* function loss (22). Some researchers suggest that the combination of *SST* and *SSTR2* may affect the TME, but clinical evidence is lacking (25). Our study investigated the association between *SSTR2* expression and immune signatures. We found that high *SSTR2* expression groups have higher median immune cell infiltration scores and immune function pathway scores compared with low *SSTR2* expression groups, suggesting that high *SSTR2* expression is associated with better immune infiltration, activation, and maintenance. This may account for the improvement of OS in patients with high *SSTR2* expression in ICIs treated cohorts. Currently, the *SSTR2* effects on TME are not well-investigated, and our study could bring new insights to the role that *SSTR2* plays in TME.

Previous studies have developed various biomarkers that can screen patients who will respond to ICIs. Currently, *PD-L1* IHC and TMB are the major biomarkers, however, they have limitations. For example, there are some patients without *PD-L1* expression that can still respond to ICIs, while others with high *PD-L1* expression do not benefit from ICIs treatment (40, 41). TMB serves as a surrogate indicator of tumor neoantigen and has no standard calculation formula or cut-off value (17). Furthermore, there are controversies regarding the use of TMB in ICIs prognosis in recent studies (42). MSI is another biomarker approved for ICIs clinical practice. However, intertumoral heterogeneity and intratumoral heterogeneity, which exist widely in tumors, interfere with the action of MSI. Moreover, multiple gene signatures, including immune cell infiltration scores and IFN-γ signatures, are currently not available for clinical use due to their high cost (43, 44). Our study demonstrates that high expression of *SSTR2* is associated with high objective response rate and longer OS in ICIs treated cohorts. This indicates that *SSTR2* could be a potential biomarker for response to ICIs.

This study has several limitations. First, limited information about *SSTR2* mutation in ICIs treated cohorts prevented us from investigating the effect of functional *SSTR2* mutation on ICIs treated patient prognosis. More molecular studies including cell line and animal models are needed to clarify the underlying mechanism of the effect of *SSTR2* on TME. Second, limited IHC results in a single cancer type may cause statistical bias; pooled analysis and consistent results from multiple cancers could minimize this bias. Third, as patient numbers in Nathanson cohort and Miao Cohort were limited, we were not able to perform statistical tests for their responders.

Our study explored the association between *SSTR2* expression and immune signatures with ICIs treatment efficacy across multiple cancers. We found that high *SSTR2* expression in patients had enduring clinical benefits and was associated with longer OS and activated immunity. Therefore, *SSTR2* could be a novel potential predictive biomarker for identifying patients who may benefit from ICIs treatment.

## Data Availability

The original contributions presented in the study are included in the article/Supplementary Material, further inquiries can be directed to the corresponding authors.

## Supplementary Material

Supplementary Figure S1 The alteration rate of SSTR2 in TCGA database.

Supplementary Figure S2 The survival analysis of SSTR2 in TCGA pooled cohort.

## Glossary

ACC: adrenocortical carcinoma
BLCA: bladder urothelial carcinoma
BRCA: breast invasive carcinoma
CESC: cervical squamous cell carcinoma and endocervical adenocarcinoma
CHOL: cholangiocarcinoma
COAD: colon adenocarcinoma
DLBC: lymphoid neoplasm diffuse large B-cell lymphoma
ESCA: esophageal carcinoma
GBM: glioblastoma multiforme
HNSC: head and neck squamous cell carcinoma
KICH: kidney chromophobe
KIRC: kidney renal clear cell carcinoma
KIRP: kidney renal papillary cell carcinoma
LAML: acute myeloid leukemia
LGG: brain lower grade glioma
LIHC: liver hepatocellular carcinoma
LUAD: lung adenocarcinoma
LUSC: lung squamous cell carcinoma
MESO: mesothelioma
OV: ovarian serous cystadenocarcinoma
PAAD: pancreatic adenocarcinoma
PCPG: pheochromocytoma and paraganglioma
PRAD: prostate adenocarcinoma
READ: rectum adenocarcinoma
SARC: sarcoma
SKCM: skin cutaneous melanoma
STAD: stomach adenocarcinoma
TGCT: testicular germ cell tumors
THCA: thyroid carcinoma
THYM: thymoma
UCEC: uterine Corpus Endometrial Carcinoma
UCS: uterine carcinosarcoma
UVM: uveal melanoma.
