# Supplementary figures and images for: Somatostatin Receptor 2: A Potential Predictive Biomarker for Immune Checkpoint Inhibitor Treatment
Source: Pathol Oncol Res. 2022 Feb 21;28:1610196. doi: 10.3389/pore.2022.1610196 (PMC8898825; doi:10.3389/pore.2022.1610196)

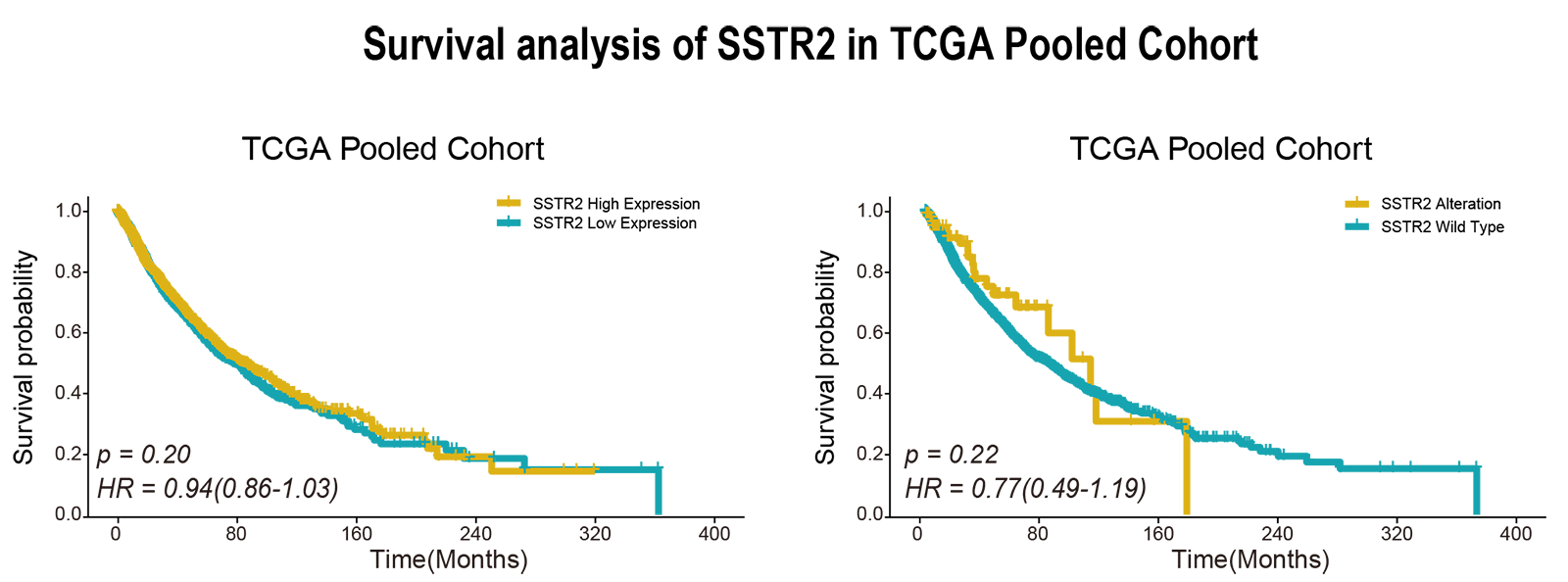

Supplement: Supplementary file 1 [file Image2.TIF]

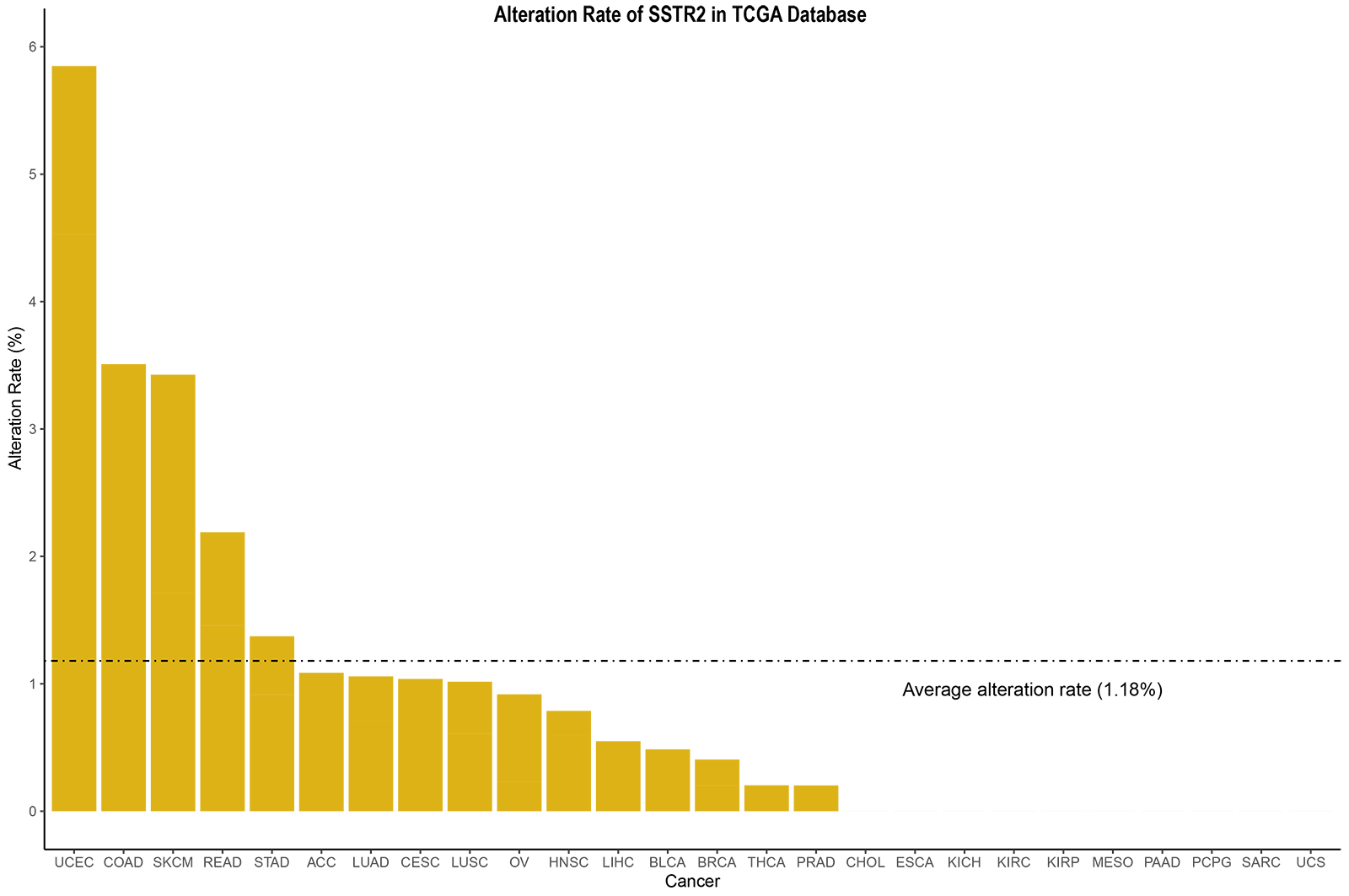

Supplement: Supplementary file 2 [file Image1.TIF]
